# Sudden Unexpected Death in Epilepsy: Addressing the Challenges

**DOI:** 10.1007/s11910-014-0502-4

**Published:** 2014-10-10

**Authors:** W. Henry Smithson, Brigitte Colwell, Jane Hanna

**Affiliations:** 1 Department of General Practice, University College Cork Ireland, Western Gateway Building, Western Road, Cork, Ireland; 2 SUDEP Action, Oxford, UK; 3 Academic Unit of Primary Medical Care, University of Sheffield Samuel Fox House, Northern General Hospital, Herries Road, Sheffield, UK

**Keywords:** SUDEP, Disclosure, Mechanisms, Prevention, Risk reduction

## Abstract

Epilepsy is associated with a higher rate of premature death than the general population, and the commonest cause of epilepsy mortality is sudden unexpected death in epilepsy (SUDEP). It is difficult to quantify because of the variable reporting of this cause of death. Death occurs due to autonomic deregulation of cardio-respiratory pathways as a result of seizures. Measures to reduce cardio-respiratory dysfunction are discussed together with the importance of seizure control in preventing SUDEP. The role of seizure detection devices, antiepileptic drugs and the importance of providing information about SUDEP to people with epilepsy are highlighted. There is increasing interest in SUDEP and some current initiatives are discussed.

## Introduction

Epilepsy is the most common serious neurological condition, affecting almost 60 million people worldwide [1]. People with epilepsy may be two to three times more at risk of dying prematurely as a result of their illness when compared to a normal population [2], and sudden death in people with epilepsy is over 20 times more frequent than in the general population [3].

Sudden unexpected death in epilepsy (SUDEP) is an uncommon but tragic consequence of epilepsy. SUDEP has received considerable examination over the last 10 years with a revision of the definition, an attempt to more accurately estimate the true incidence, a better understanding of the mechanisms and risk factors leading to death, and how the risk of SUDEP is discussed with patients and families. Significant funding is currently available from the National Institute for Health with SUDEP being the subject of a Centre without Walls call in 2014. This followed the research recommendations of The National Institute for Health/National Institute for Neurological Disease and Stroke Workshop on SUDEP in 2011 [4]. There are significant challenges associated with research into sudden death in epilepsy. This paper will outline how SUDEP is now defined, the difficulties in case ascertainment as a result of variable reporting and the difficulties this imposes on estimates of incidence. The mechanisms of SUDEP are described, and how improved understanding of why SUDEP occurs may lead to effective risk reduction strategies and finally how the risk may be reduced.

## Defining SUDEP

SUDEP was first defined nearly 20 years ago and since then, two complimentary definitions have been in use [5, 6]. It is important to unify the definition to reduce ambiguity and to retrieve cases that would not have been studied using the earlier definition. It is proposed that the term ‘unexpected’ should be used rather than ‘unexplained’ that SUDEP should be categorised where appropriate and an additional category of SUDEP plus should be designated when it is likely that a pre-existing condition could have contributed to death. To be considered SUDEP, the death should have occurred within an hour of the terminal event and status epilepticus as an exclusion criterion is when the seizure duration is more than 30 min [7•]. Definite SUDEP can be used when a post-mortem examination shows no definite cause of death, probable SUDEP when post-mortem examination is not performed but other criteria are fulfilled and possible SUDEP is applied when a competing cause of death is present. Near SUDEP is sometimes used in cases where death is likely to have occurred if resuscitation or other interventions had not been applied [4–6, 7•].

## Reporting SUDEP

SUDEP is the main cause of death in individuals with epilepsy but the term is underused in death certification [8]. The post-mortem examination of sudden death from any cause is problematic and requires a careful and detailed interrogation of families and witnesses to identify past medical history, symptoms, medication use [9] and circumstances leading up to death. This is particularly important in the light of sub-optimal reporting.

A UK study examined 612 death entries (60 % male), with a median age of 35. Four hundred and ninety-eight had undergone post-mortem examination, of which 44 stated that SUDEP was the cause of death and 292 were considered probable SUDEP. They found 69 were attributable to status epilepticus, with a further 71 epilepsy-related deaths. Close examination of the documentation suggested that nearly half of the cases attributed to status epilepticus are more appropriately classified as SUDEP and that status epilepticus as a cause of death should only be recorded if there is a documented history of uncontrolled seizures. The conclusion was that correct certification of death is essential to provide accurate data on SUDEP and other epilepsy deaths and to ensure a more accurate picture of the prevalence of SUDEP [10]. Similar problems were identified in the USA. A retrospective evaluation of forensic autopsy cases 2007–2009 was conducted in the State of Maryland, which has 15 medical examiners (MEs). One hundred and four sudden unexpected deaths which were directly or indirectly caused by epilepsy or seizure disorder were found, of which 74 met the definition of SUDEP. However, of the 74 cases, seizure disorder was listed as the primary cause of death in 65 cases and epilepsy as the primary cause in 1, with SUDEP being listed at the primary cause of death in only 8 cases. It was found that despite 14/15 MEs acknowledging SUDEP as a valid diagnosis, only two used SUDEP as an official cause of death, with the majority preferring to use seizure disorder or epilepsy rather than a term that was felt to imply some uncertainty around the diagnosis [11].

A survey about the use of the diagnosis of SUDEP as a cause of death was administered to all coroners and medical examiners in the USA. While 83.5 % of pathologists acknowledged SUDEP as a valid diagnosis if no cause of death is found at autopsy, only 22.9 % went on to use SUDEP in cases eligible for the diagnosis, irrespective of their educational background, location, autopsy rate or number of seizure cases seen and conclude that SUDEP appears to be underused as a final diagnosis by MEs throughout the USA [8].

If the correct cause of death can be reported on certificates, the true incidence of SUDEP may be found to be much higher than currently reported [12]. Following the increasing awareness of SUDEP, in 2013, two American states (New Jersey and Illinois) signed SUDEP measures into law requiring medical examiners to find out about potential history of epilepsy as part of the autopsy. It remains to be seen if this example is followed by other states but the need for better education concerning SUDEP is clear [3].

## Incidence of SUDEP

In population-based studies, the stated incidence range from 0.09 to 2.31/1000 patient-years [13] and this rate is higher in patients with refractory epilepsy awaiting assessment for a surgical programme [14] at 9.3/1000 patient-years. A study of a cohort of children diagnosed with epilepsy in 1964 from Finland, where there is a high rate of autopsy, observed over 40 years showed a rate of SUDEP of 7 % overall and 12 % excluding patients in remission [15]. A prospective audit of 4578 patients with epilepsy in the mid-west of the USA over 5 years collected data from 16,463 patient-years. One hundred and eleven patients died in this period of which 10 were judged to be definite SUDEP and 10 probable SUDEP. This gave an overall incidence of SUDEP of 1.21/1000 patient-years with the highest incidence in the 50–59 age range [16].

A retrospective study of mortality in Epilepsy Monitoring Units (EMU) demonstrated a total SUDEP incidence of 5 · 1 (95 % CI 2 · 6–9 · 2) per 1000 patient-years and suggests that SUDEP could be associated with suboptimum supervision and antiepileptic drug withdrawal [17•]. This study identified 160 EMU in Europe, Australia, New Zealand and Israel and achieved a 92 % survey response rate with data collected from an estimated 133,788 video electroencephalogram (VEEG) and analysed in subgroups of age and reason for monitoring. There was only one SUDEP/near SUDEP case in children and the total rate of adult SUDEP/near SUDEP was 7.9 (4.6–12.7), 9.7 (5.2–16.7) in pre-surgical assessment and 4.9 (4.6–12.7) per 1000 patient-years in ‘other VEEG’ monitored cases [17•].

Given the complex judgements required in defining definite/probable/possible/near SUDEP and the variable standards of post mortem investigation and death certification, it is difficult to accurately calculate the incidence of SUDEP. To draw out more concrete data, a systematic review of incidence papers is currently being carried out by an international group convened by the American Association of Neurologists (AAN) to review all available data and preliminary results from this review show the incidence of SUDEP to depend on type and severity of epilepsy with an overall incident rate range for the general epilepsy population, including children and adults, estimated at 0.7–1.5 per 1000 patient-years, inclusive of refractory patients (personal communication Harden). This review should provide useful sub-group analysis to inform future work in this area.

## Reducing the Risk of SUDEP

Reducing the risk of SUDEP requires an understanding of why it happens, how it happens, how to detect and address prodromal events and how to control seizures. There are, however, no clear cut answers.

### Understanding the Mechanisms Underlying SUDEP

The substantial work published on identifying a mechanism suggests that there are overlapping cardiac, respiratory and autonomic domains but no clear evidence for pre-existing pathway or structural abnormalities [18]. Demonstrating this overlap, the MORTEMUS study sheds new light on a previously unrecognised pattern (termed early postictal neurovegetative breakdown) from observed SUDEP with initial generalized tonic-clonic seizure (GTCS) triggering a short period of normal or increased heart and respiratory rates then severe bradycardia and central apnoea with post-ictal EEG suppression that is fatal in a third of cases. In those who survive the initial impact, there is transient improvement of cardiac function but associated with abnormal and ineffective respiration that can be aggravated by the prone position before terminal apnoea followed by terminal asystole [17•].

Autonomic dysfunction resulting in poor homeostatic responses has also been implicated in sudden infant death syndrome (SIDS) with overstimulation of the adenosine receptors [19] and a defect in 5-HT brainstem-mediated control of respiratory and autonomic regulation [20]. In animal models, progressive neuronal loss as a result of convulsions has been noted, for example, in the nucleus tractus solitarius (NTS) (the afferent autonomous gatekeeper) and sclerosis of the lateral amygdaloid nuclei [21], and may play a role in impairing the integrative functions of the NTS resulting in poor homeostatic responses during seizures and leading to SUDEP [22]. Dysfunction of the 5-HT axis can lower the seizure threshold and increase the risk of depression and sudden death, and this pathophysiological mechanism may be shared with SIDS. The converse is true in that an increase in extracellular 5-HT may raise the seizure threshold [23]. 5-HT affects many other cerebral functions, including arousal, thermoregulation, circadian rhythms, anxiety, pain, aggression and respiratory control, and may be a factor in the similarities between SUDEP and SIDS [24]. However, research is needed to translate these findings perhaps into the development of agents that enhance serotonin (5-HT) to reduce seizures and SUDEP. One promising avenue is the selective serotonin reuptake inhibitors (SSRI). Fluoxetine was found to reduce respiratory depression in patients undergoing telemetry [25] and reduction of respiratory depression in seizure induced mice [26]. It may be that agents such as fluoxetine can reduce the risk of SUDEP [27].

Cardiac dysfunction may also provide clinical markers for SUDEP [28]. In particular, reduced heart rate variability (HRV) may be a predisposing factor for SUDEP [29]. A re-analysis of this study showed that there are differences between high-frequency HRV [30] and a further study of 19 individuals showed an association between HRV and SUDEP risk [31] and this association with progressive deterioration of HRV was noted in a case report [32].

Heart rate variability leads strength to the proposal that all patients with epilepsy should have an assessment of their cardiac function to rule out channelopathies. There is some support for the hypothesis of single ion channel mutation (KCNA1 gene) that could underlie both epilepsy and cardiac arrhythmias [3]. The association between epilepsy and cardiac function suggest that omega 3 fatty acids may be of benefit given a number of studies having shown the consumption of fatty fish may reduce the number of sudden cardiac deaths, and a number of animal and clinical studies suggest that omega-3 fatty acids may be useful in the prevention and treatment of epilepsy [33].

### Seizure Detection Devices

Seizure detection systems can be used to alert observers to intervene to reduce the effects of seizures. The most sophisticated monitoring system combines video and EEG recording. This complex system is labour intensive and usually limited to in-patient hospital use. There is a growing demand for monitoring devices that can be used in the home to detect seizures to enable arousal interventions to prevent SUDEP. A number of devices are available and may measure motor activity [34], measure cardiovascular or respiratory changes, detect associated autonomic changes through electro-dermal activity, audio detection, temperature, ocular and eyelid movements, body pressure or moisture [1].

Devices need to be able to reliably detect seizure activity, but if the device gives false-positive alarms that affects sleep, it becomes unacceptable to the individual and observers. Given the individual nature of seizures, the most effective way to detect seizures may be to use a combination of systems. While acknowledging the gold standard for monitoring epileptic seizures continues to be video/EEG, which takes place in controlled environments such as Epilepsy Monitoring Units (EMUs), there are other body signals that can be monitored and the response in terms of monitoring and detection systems that are available. There are a range of body movements that can be monitored to detect seizure activity, but that the detection systems must be non-invasive, multi-modal and appropriate to use in a domestic setting [1] .

A prospective study of a flexible movement monitor tested on children over 2 years with a monitor designed to fit under a mattress and attached to a monitor [35]. The study team tested the monitor in a hospital setting with concurrent EEG, cardiopulmonary and nurse monitoring. Seventy-eight seizures were recorded by EEG videos in 45 patients. The monitor captured 30 % of all seizure events, 54 % of events while sleeping and 85 % of GTCS while sleeping. The alarm was not sensitive to seizures with no rhythmical movements. A similar mattress monitor study in patients aged 13–65 years with tonic-clonic seizures demonstrated the need for individual calibration [36, 37]. The potential benefit of a mattress detection device for GTCS in 79 patients (after excluding 28 patients because of a faulty sensor or inaccurate recording of data) showed a PPV of 0.43 and NPV of 0.98.

A wrist-worn wireless accelerometer has been shown to be useful in detecting GTCS with a low false-positive rate [37]. The authors suggest further work is required on a larger group of patients in an ambulatory setting. However, results were disappointing in a prospective study of two bed alarms for detection of nocturnal seizures with only one GTCS detected out of 15 seizures while sleeping [38]. Given the search for effective home-based seizure monitoring and the need for an individualised multi-modal system, convincing evidence for non-EEG systems is still awaited [1].

### Controlling Seizures with Antiepileptic Drugs

Antiepileptic drugs (AEDs) remain the treatment of choice in controlling seizures [39] and thereby eliminating the risk of SUDEP. However, while there have been studies that raised concerns about specific drugs and therapeutic regimes, the evidence is unconvincing [16, 40]. Indeed, a meta-analysis of 97 papers investigating any AED adjunct treatment of drug-resistant epilepsy suggests that adjunctive AED at efficacious doses reduce the incidence of definite or probable SUDEP by more than seven times compared to placebo and so provides evidence in favour of active treatment for patients with refractory epilepsy [41]. A commentary on the issues raised by this important paper [42] reinforces the safety of adjunct therapies and the risks to patients in placebo arms of comparative studies.

AEDs are most effective when they are taken as prescribed. Non-adherence to medicines leads to reduced drug levels and has been implicated in sub-optimal management [43] and SUDEP [44, 11]. Non-adherence to medicines has been identified as a factor in cases of SUDEP [45, 46]. Medicine non-adherence is a dynamic and variable behaviour and so patients’ use of medicine should be reviewed on a regular basis [47]. To do this, the patient needs to be a partner in decisions about treatment and this requires information about the impact of the condition. When patients are aware of the risks associated with epilepsy including SUDEP, they are given an opportunity to take decisions to reduce that risk, particularly in how they manage their epilepsy and take their AED [48, 49•].

### Optimising Self-Management by Understanding the Risks Associated with Epilepsy

For many years, there was reluctance by some health professionals to talk openly about SUDEP. However, the weight of opinion is shifting to full disclosure. A call for openness on SUDEP, encouraging early discussion of risk, was backed by an international expert panel and 14 international epilepsy organisations at the 30th International Epilepsy Congress in Montreal held in 2013 (www.sudep.org/article/sudepactionleadscallforopenness2013).

In response to calls for disclosure and discussion, many health professionals are unsure how to respond. This suggests that the philosophy of self-management and informed decision-making have not been universally adopted into epilepsy care. The SUDEP debate has injected some urgency into discussion on how epilepsy education generally should take place, something which is long overdue [50]. To assist health professionals to respond, a report from the joint working party of the American Epilepsy Society and Epilepsy Foundation summarises risks and preventative strategies [51]. In particular, information about SUDEP is important for patients at risk of AED non-adherence and for those who are candidates for surgery and can be reassuring for patients with well-controlled epilepsy who are at low risk.

## Discussion

The challenge of preventing sudden death from epilepsy is currently receiving more attention than previously with increasing numbers of research publications and conference posters relating to SUDEP. There is a SUDEP Special Interest Group within the AES, and Partners Against Mortality in Epilepsy (PAME) held their second international meeting in Minneapolis USA in 2014. This conference attracted participants from across the world and included bereaved families, researchers and clinicians. SUDEP is now a subject for disclosure and discussion, and in recognition of this more open approach, SUDEP Registries have been established for reporting by professionals (www.sudep.org/) and from families (http://epilepsydeathsregister.org/) (www.sudepaware.org/) (www.epilepsyireland.org/) to provide a wider collection of data concerning cases.

By revisiting the definition, it should provide clearer guidance for certificating SUDEP as the cause of death and for epidemiologists involved in population-based studies by standardising the datasets. Only by doing this will the true incidence be uncovered. The progress made in describing mechanisms of death allows the term unexplained to be discarded. The element of doubt is incorporated in the definition with definite, probable and possible being included. Near-SUDEP is most likely to be recorded in events observed and remedied in EMU but it must not be forgotten that although SUDEP is more common in patients with refractory epilepsy, it can and does occur in patients viewed as being at low risk. These cases will only be recorded if good post-mortem practice is more widespread, and it is encouraging that two states in the USA have made it mandatory for MEs to gather information about epilepsy. This essential instruction to coroners and MEs must become commonplace.

The precise mechanism of SUDEP is still unknown but the MORTEMUS study gives a novel insight into the progression of events described as post-ictal neurovegetative breakdown. Cardiac monitoring, prevention of respiratory depression and cardiac function by manipulation of the autonomic pathways by SSRI co-prescribing and omega 3 supplements may be valuable avenues for exploration.

In those people where seizure control is unachievable, then, seizure monitoring can be undertaken. The methods must be individualised and sensitive enough to avoid poor compliance due to false alarms and acceptable for home use. There is no single best device and effective monitoring may require a multi-modal approach. This presents a challenge of affordability and complexity.

It is reassuring that a meta-analysis of adjunct AED therapy shows the benefit of polytherapy in efficacious doses. Previous evidence about the association of adjunct AED and SUDEP seems to be the result of epilepsy severity rather than caused by polytherapy. Of concern is the risk of SUDEP in patients attending EMU who are in the placebo phase of the assessment and the necessarily rapid AED withdrawal in these patients. If evidence relating to the safety of slower tapering of dose is available then a case for longer in-patient stay could be made.

Where good seizure control is possible, there is a need for continuing and open discussion about decisions in respect of management, particularly in regard to medicines non-adherence. This is a common behaviour and yet not fully discussed or assessed during the consultation. This could be a key to reducing risk and is made easier with the information available from not-for-profit organisations. The development of registers of epilepsy-related death offers an opportunity for something positive to come out of such a tragic event and all involved in the care of people with epilepsy may wish to signpost the bereaved to these important initiatives.

## References

[1] Van de Vel A, Cuppens K, Bonroy B, Milosevic M, Jansen K, Van Huffel S (2013). Non-EEG seizure-detection systems and potential SUDEP prevention: state of the art. Seizure.

[2] Scorza FA, Arida RM, Terra VC, Cavalheiro EA (2010). What can be done to reduce the risk of SUDEP?. Epilepsy Behav: E&B.

[3] Donner EJ (2011). Explaining the unexplained; expecting the unexpected: where are we with sudden unexpected death in epilepsy?. Epilepsy Currents / Am Epilepsy Soc.

[4] Hirsch LJ, Donner EJ, So EL, Jacobs M, Nashef L, Noebels JL (2011). Abbreviated report of the NIH/NINDS workshop on sudden unexpected death in epilepsy. Neurology.

[5] Nashef L (1997). Sudden unexpected death in epilepsy: terminology and definitions. Epilepsia.

[6] Annegers JF (1997). United States perspective on definitions and classifications. Epilepsia.

[7.•] Nashef L, So EL, Ryvlin P, Tomson T (2012). Unifying the definitions of sudden unexpected death in epilepsy. Epilepsia.

[8] Schraeder PL, Delin K, McClelland RL, So EL (2006). Coroner and medical examiner documentation of sudden unexplained deaths in epilepsy. Epilepsy Res.

[9] de la Grandmaison GL (2006). Is there progress in the autopsy diagnosis of sudden unexpected death in adults?. Forensic Sci Int.

[10] Langan Y, Nashef L, Sander JW (2002). Certification of deaths attributable to epilepsy. J Neurol Neurosurg Psychiatry.

[11] Zhuo L, Zhang Y, Zielke HR, Levine B, Zhang X, Chang L (2012). Sudden unexpected death in epilepsy: evaluation of forensic autopsy cases. Forensic Sci Int.

[12] Lathers CM (2009). Epilepsy and sudden death: personal reflections and call for global action. Epilepsy Behav: E&B.

[13] Tomson T, Nashef L, Ryvlin P (2008). Sudden unexpected death in epilepsy: current knowledge and future directions. Lancet Neurol.

[14] Dasheiff RM (1991). Sudden unexpected death in epilepsy: a series from an epilepsy surgery program and speculation on the relationship to sudden cardiac death. J Clin neurophys : Off Publ Am Electroencephalogr Soc.

[15] Sillanpää M, Shinnar S (2010). Long-term mortality in childhood-onset epilepsy. N Engl J Med.

[16] Walczak TS, Leppik IE, D’Amelio M, Rarick J, So E, Ahman P (2001). Incidence and risk factors in sudden unexpected death in epilepsy: a prospective cohort study. Neurology.

[17.•] Ryvlin P, Nashef L, Lhatoo SD, Bateman LM, Bird J, Bleasel A (2013). Incidence and mechanisms of cardiorespiratory arrests in epilepsy monitoring units (MORTEMUS): a retrospective study. Lancet Neurol.

[18] So EL (2008). What is known about the mechanisms underlying SUDEP?. Epilepsia.

[19] Shen HY, Li T, Boison D (2010). A novel mouse model for sudden unexpected death in epilepsy (SUDEP): role of impaired adenosine clearance. Epilepsia.

[20] Patterson D (2009). Medullary serotonin defects and respiratory dysfunction in sudden infant death syndrome. Respir Physiol Neurobiol.

[21] Thom M, Griffin B, Sander JW, Scaravilli F (1999). Amygdala sclerosis in sudden and unexpected death in epilepsy. Epilepsy Res.

[22] Tolstykh GP, Cavazos JE (2013). Potential mechanisms of sudden unexpected death in epilepsy. Epilepsy Behav: E&B.

[23] Richerson GB, Buchanan GF (2011). The serotonin axis: shared mechanisms in seizures, depression, and SUDEP. Epilepsia.

[24] Sowers LP, Massey CA, Gehlbach BK, Granner MA, Richerson GB (2013). Sudden unexpected death in epilepsy: fatal post-ictal respiratory and arousal mechanisms. Respir Physiol Neurobiol.

[25] Bateman LM, Li C-S, Lin T-C, Seyal M (2010). Serotonin reuptake inhibitors are associated with reduced severity of ictal hypoxemia in medically refractory partial epilepsy. Epilepsia.

[26] Faingold CL, Tupal S, Randall M (2011). Prevention of seizure-induced sudden death in a chronic SUDEP model by semichronic administration of a selective serotonin reuptake inhibitor. Epilepsy Behav: E&B.

[27] Boison D (2012). A breather for SUDEP. Epilepsy Curr.

[28] Surges R, Sander JW (2012). Sudden unexpected death in epilepsy: mechanisms, prevalence, and prevention. Curr Opin Neurol.

[29] Surges R, Henneberger C, Adjei P, Scott CA, Sander JW, Walker MC. Do alterations in inter-ictal heart rate variability predict sudden unexpected death in epilepsy? Epilepsy Research. 2009;87(2–3):277–80. 10.1016/j.eplepsyres.2009.08.008 19747799

[30] DeGiorgio CM, DeGiorgio AC (2010). SUDEP and heart rate variability. Epilepsy Res.

[31] DeGiorgio CM, Miller P, Meymandi S, Chin A, Epps J, Gordon S (2010). RMSSD, a measure of vagus-mediated heart rate variability, is associated with risk factors for SUDEP: the SUDEP-7 Inventory. Epilepsy Behav: E&B.

[32] Rauscher G, DeGiorgio AC, Miller PR, DeGiorgio CM (2011). Sudden unexpected death in epilepsy associated with progressive deterioration in heart rate variability. Epilepsy Behav: E&B.

[33] Scorza FA, Cysneiros RM, Arida RM, Terra-Bustamante VC, de Albuquerque M, Cavalheiro EA (2008). The other side of the coin: beneficiary effect of omega-3 fatty acids in sudden unexpected death in epilepsy. Epilepsy Behav : E&B.

[34] Conradsen I, Moldovan M, Jennum P, Wolf P, Farina D, Beniczky S (2013). Dynamics of muscle activation during tonic-clonic seizures. Epilepsy Res.

[35] Poppel KV, Fulton SP, McGregor A, Ellis M, Patters A, Wheless J (2013). Prospective study of the Emfit Movement Monitor. J Child Neurol.

[36] Carlson C, Arnedo V, Cahill M, Devinsky O (2009). Detecting nocturnal convulsions: efficacy of the MP5 monitor. Seizure.

[37] Beniczky S, Polster T, Kjaer TW, Hjalgrim H (2013). Detection of generalized tonic-clonic seizures by a wireless wrist accelerometer: a prospective, multicenter study. Epilepsia.

[38] Fulton S, Poppel KV, McGregor A, Ellis M, Patters A, Wheless J (2013). Prospective study of 2 bed alarms for detection of nocturnal seizures. J Child Neurol.

[39] Langan Y, Nashef L, Sander JW (2005). Case-control study of SUDEP. Neurology.

[40] Walczak T (2003). Do antiepileptic drugs play a role in sudden unexpected death in epilepsy?. Drug Safety : Intl J Med Toxicol Drug Exp.

[41] Ryvlin P, Cucherat M, Rheims S (2011). Risk of sudden unexpected death in epilepsy in patients given adjunctive antiepileptic treatment for refractory seizures: a meta-analysis of placebo-controlled randomised trials. Lancet Neurol.

[42] Hesdorffer DC, Tomson T (2011). Adjunctive antiepileptic drug therapy and prevention of SUDEP. Lancet Neurol.

[43] Smithson WH, Hukins D, Buelow JM, Allgar V, Dickson J (2013). Adherence to medicines and self-management of epilepsy: a community-based study. Epilepsy Behav: E&B.

[44] Lathers CM, Koehler SA, Wecht CH, Schraeder PL (2011). Forensic antiepileptic drug levels in autopsy cases of epilepsy. Epilepsy Behav: E&B.

[45] Bellon M, Panelli R, Rillotta F. Exploring the experiences and needs of people bereaved by epilepsy: results from an online Australian survey (poster). 10th Asian and Oceanian Congress, Singapore. 2014.

[46] Kennelly C, Riesel J. Sudden Death and Epilepsy: The views and experiences of bereaved relatives and carers. Epilepsy Bereaved. 2002. Available from: https://www.sudep.org/sudden-death-and-epilepsy.

[47] Smithson WH, Hukins D, Colwell B, Mathers N (2012). Developing a method to identify medicines non-adherence in a community sample of adults with epilepsy. Epilepsy Behav.

[48] Paschal AM, Rush SE, Sadler T (2014). Factors associated with medication adherence in patients with epilepsy and recommendations for improvement. Epilepsy Behav: E&B.

[48.•] Chapman SC, Horne R, Chater A, Hukins D, Smithson WH (2014). Patients’ perspectives on antiepileptic medication: relationships between beliefs about medicines and adherence among patients with epilepsy in UK primary care. Epilepsy Behav: E&B.

[50] Prinjha S, Chapple A, Herxheimer A, McPherson A (2005). Many people with epilepsy want to know more: a qualitative study. Fam Pract.

[51] So EL, Bainbridge J, Buchhalter JR, Donalty J, Donner EJ, Finucane A (2009). Report of the American Epilepsy Society and the Epilepsy Foundation joint task force on sudden unexplained death in epilepsy. Epilepsia.

